# FUSTA: leveraging FUSE for manipulation of multiFASTA files at scale

**DOI:** 10.1093/bioadv/vbac091

**Published:** 2022-11-29

**Authors:** Franklin Delehelle, Hugues Roest Crollius

**Affiliations:** Département de biologie, Institut de Biologie de l’ENS (IBENS), École normale supérieure, CNRS, INSERM, Paris 75005, France; Département de biologie, Institut de Biologie de l’ENS (IBENS), École normale supérieure, CNRS, INSERM, Paris 75005, France

## Abstract

**Motivation:**

FASTA files are the *de facto* standard for sharing, manipulating and storing biological sequences, while concatenated in multiFASTA they tend to be unwieldy for two main reasons: (i) they can become big enough that their manipulation with standard text-editing tools is unpractical, either due to slowness or memory consumption; (ii) by mixing metadata (headers) and data (sequences), bulk operations using standard text streaming tools (such as sed or awk) are impossible without including a parsing step, which may be error-prone and introduce friction in the development process.

**Results:**

Here, we present FUSTA (FUse for faSTA), a software utility which makes use of the FUSE technology to expose a multiFASTA file as a hierarchy of virtual files, letting users operate directly on the sequences as independent virtual files through classical file manipulation methods.

**Availability and implementation:**

FUSTA is freely available under the CeCILL-C (LGPLv3-compatible) license at https://github.com/delehef/fusta.

**Supplementary information:**

Supplementary data are available at *Bioinformatics Advances* online.

## 1 Introduction

Originally developed for the FASTA program (Pearson and Lipman, 1988), the FASTA format and its multiFASTA extension, containing multiple sequences in a single file, have become the *de facto* standards for sharing, processing and storing DNA, RNA and protein sequences in the bioinformatics community. This success reflects the intrinsic qualities of this format: FASTA files are self-contained, easily readable by both humans and machines, and their use of a text format relying only on the ASCII standard facilitates their diffusion without concerns for neither the encoding nor the management of related bytes in memory on the underlying systems (notably endiannes). Although their encoding, using a whole byte for only a handful of bases, is rather sparse, this limit is easily mitigated by efficient compression tools or intermediate storage formats (Kryukov *et al.*, 2020). However, the fine-grained manipulation of large multiFASTA files is often a hurdle, as attested by the plethora of scripts developed to tackle specific tasks such as sequences extraction, single-line conversion or up- and down-case conversion; all of them reimplementing FASTA parsing to some extent.

Difficulties generally stem from two root causes. First, multiFASTA files can become quite large (up to dozens of gigabytes), and most text editors are not designed to smoothly manipulate such large files, hindering the classical [open file → region selection → transformation → save file] workflow. Second, the interleaving of metadata (headers) and data (sequences) prevents the use of file-wide transformations (using either text editors or command line utilities), as those could inadvertently affect headers as a side-effect.

These issues have been partially solved by the development of FASTA index files, which keep track of the precise locations of every sequence in the multiFASTA file, and by other programs dedicated to FASTA files manipulation by implementing a set of operations on them (Hunt, 2012; Jackman, 2012; Li, 2012; Shen *et al.*, 2016).

However, these approaches result in top-down, fully integrated software workflows, which have to reimplement every text-manipulation operation in their own context of operation, without being able to easily leverage commodities such as standard POSIX piping or powerful text editors.

Another approach has been the development of language-specific libraries, for instance for Python (Cock *et al.*, 2009), Perl (Stajich *et al.*, 2002) and Rust (Köster, 2016). These approaches, while extremely flexible, are however limited to a single programming language, and are not easily transferable to another language ecosystem.

Here, we present FUSTA (**FUS**E for FAS**TA**), a new approach to the problem. By making a standard multiFASTA file appear as an arborescence of files representing its individual sequences and metadata, FUSTA let users leverage the whole existing ecosystem of text-manipulation tools without having to worry about technical concerns.

## 2 The FUSTA tool

FUSE (**F**ilesystem in **USE**rspace) is an OS interface available in Linux, macOS and the BSD family, by which userspace programs may present arbitrary data as a hierarchy of pseudo-files and directories within a given directory, called the *mount point*. FUSTA is a command-line tool (thus requiring some degree of familiarity with UNIX-like operating systems) implementing a FUSE module, exposing a single multiFASTA file as a hierarchy of virtual files and folders reflecting the structure and content of the mounted file, letting the user transparently read, edit, and remove contained sequences as if they were standing independently, these operations being transparently applied to the original multiFASTA file. FUSTA must be invoked with a multiFASTA file as an argument, which will be exposed (or mounted) in a directory (the mount point), which will serve as a base point for further operations. FUSTA will populate this folder with the labels.txt, infos.txt, and infos.csv files, and the seqs/, fasta/, append/ and get/ folders. All the following examples assume the user mounted a multiFASTA file and that the current directory is the mount point.

FUSTA has been designed to operate transparently on a vast variety of multiFASTA files, while preserving the original formatting. Therefore, FUSTA can operate on gapped files (such as alignment result) and wrapped files (without concern for the size or the homogeneity of the padding), files containing empty sequences, and support any character within the sequence themselves, including but not restricted to IUPAC alphabet. The only requirements are (i) lines must be using UNIX delimiters (\n), (ii) each sequence must have an ID and (iii) sequence IDs must be valid filename characters in the OS where FUSTA is used.

Although short examples of use will be provdided in the following sections, more thorough ones are provided in the Supplementary Data.

### 2.1 Labels and general informations

The labels.txt virtual text file is a read-only register of all the headers contained in the mounted file, each of them consisting of an ID and an optional description.

The human-readable infos.txt and CSV (Comma-Separated Values) -formatted infos.csv virtual text files contain general information about the mounted multiFASTA file, and a table listing general information on the sequences it contains.

### 2.2 Accessing sequences

#### 2.2.1 Raw sequences

The seqs/ directory exposes one virtual text file per sequence in the mounted FASTA file, identified by the ID of the corresponding sequence, each of them containing the raw content of the associated sequence. These files are accessible for both reading and writing—any operation altering the original multiFASTA file being reflected on it—and can thus be removed (deleting them in the mounted file), renamed (changing their ID in the mounted file), edited (accordingly changing the sequence in the mounted file), copied or moved within the folder (*i.e.* renamed) or outside of the folder (*i.e.* removed from the mounted multiFASTA file).

For instance, rmseqs/chrMT.seq would remove the chrMT sequence from the mounted multiFASTA file; and foriinseqs/*;domvseqs/${i}seqs/chr${i};done would prepend all existing sequences IDs with chr.

#### 2.2.2 FASTA-formatted sequences

The fasta/ directory contain one read-only, single-sequence FASTA file per sequence in the mounted FASTA file, allowing a fast direct access to any of them. They transparently behave as standard FASTA files, and can e.g. be copied or used as input in other programs. They are updated in real-time as the user perform state-altering operations in the virtual filesystem.

For instance, catfasta/chr{X, Y}.fa>$HOME/sex_chrs.fa would extract the sequences of the human sex chromosomes in a new multiFASTA file. Similarly, blastnmydb.db-queryfasta/seq25.fa would use the seq25 sequence from the mounted multiFASTA file as the query in a BLAST (Lipman and Pearson, 1985) search, without the need to extract and create temporary intermediate files.

### 2.3 Appending sequences

Any new FASTA or multiFASTA file written to the append/ directory, be it, *e.g.* by file copy or manually saving a file from a text editor in this folder, will not be conserved as independent files, but immediately be appended to the mounted FASTA file, and these new additions will be reflected in the other virtual files and directories. Other types of files not matching the typical multiFASTA signature (i.e. an ASCII text file whose first line starts with a closing angle bracket) that would be placed there will be ignored, and the mismatch in file types will only appear in the logs. Of note, once FASTA files are modified with FUSTA, accompanying index files required in many standard pipelines will need to be re-generated.

For instance, cp$HOME/more_sequences.faappend/ would append the sequences contained in the more_sequences.fa file to the mounted multiFASTA file.

### 2.4 Retrieving subsequences

The get/ directory let users access subsequences of sequences following the standard ID:STARTBASE-ENDBASE format, where indexing is done with a 1-based, closed interval. Although no files are apparently present in this folder, any read access to a file following this pattern in this folder will return the corresponding subsequence on the fly.

For instance, reading the get/chr17:18108706-18179802 file on the mounted human genome (hg38) would return the sequence of the MYO15A gene. With this mechanism, repeated access to random subsequences of a genome can be easily automatized, while remaining efficient thanks to the use of system-level file caches.

## 3 Implementation

FUSTA is implemented in the Rust programming language and is available for GNU/Linux, macOS, and FreeBSD under the CeCILL-C (LGPLv3-compatible) license. Depending on runtime options, accesses to the sequences in the original FASTA file is performed either through memory-mapped files (default behavior, optimized for fast repeated accesses), directly by the standard seek & read approach (reduces file cache memory consumption, avoids allowing over-allocation of memory), or just by wholly caching the underlying FASTA file in memory (very efficient for numerous random accesses, but requires as much free memory as the size of the multiFASTA file).

Altering accesses are implemented as a list of memory-cached operations, that are then propagated (on fsync and fsyncdir calls or when unmounting) on the original multiFASTA file; this approach balances latency (stemming from on-disk write operations) and memory consumption (for operation caching). The virtual files, however, are kept up to date in real-time. The cache size defaults to 500 MiB, but can be changed by the user to fit their requirements and workflow.

All the virtual files and folders exposed by FUSTA behave transparently to any process accessing them, be it through the shell or standard POSIX file manipulation primitives, e.g. from other programs or scripts. It should be noted that modifying in any way a mounted multiFASTA file breaks all guarantees regarding the file integrity.

FUSTA can be run either in foreground mode, in which it will display ongoing operations in the virtual filesystem (different verbosity levels are available); or in background mode (default behavior), where it will detach from the shell from which it has been started. In both cases, unmounting the mount point will cause FUSTA to synchronize potential pending operations, then exit. A complete list of runtime options and a short description are available with the usual help flags (or –help).

## 4 Performances

While running under the default regime (*i.e.* using mmap as a cache mechanism), FUSTA is a lightweight tool, using only enough memory to store its internal map of the mounted FASTA file, which ranges from a few hundreds kilobytes to a few dozen megabytes. Thus, FUSTA can be comfortably used on most computers, from laptops to cluster nodes.

The two main bottlenecks are (i) the initial parsing of the mounted FASTA file, which, empirically, is I/O-bound; (ii) accessing random sequence fragments, which has a fixed cost due to data passing the kernel space—user space barrier, plus multifactorial dependencies on the caching mechanism selected, the storage used (SSD or HDD), the system load, the memory pressure, the IO load, and the spatial proximity of the fragments on disk.

To compare FUSTA overhead to other alternative solutions, we ran a benchmark (Table 1) in which we measure the time required to read 1 ,000,000 random sequences from the 6.4 GiB ENSEMBL-108 proteome FASTA file (https://ftp.ensembl.org/pub/release-108/emf/ensembl-compara/homologies/Compara.108.protein_default.cds.fasta.gz), containing 4 237 432 sequences from 10 bp to 107 974 bp long, using FUSTA, Bedtools and BioPython.

**Table 1.** Resources used to extract a random set of 1 million subsequences from the ENSEMBL 108 proteome

	FUSTA/mmap	FUSTA/RAM	BedTools	BioPython
Time	1’41” (22”)	1’38” (22”)	34” (16”)	32” (41”)
Memory	1.8 GiB	11.3 GiB	1.2 GiB	11.5 GiB

*Note*: All tests were run on a 2021 MacBook, featuring an Apple M1 CPU, 16 GiB of RAM and SSD storage. Caching or indexing time is indicated in parentheses.

In this very I/O intensive benchmark, results illustrate the overhead of dating having to flow through kernel space before being delivered to user space programs, which worsen FUSTA processing time. Indeed, FUSTA with mmap caching works similarly to BedTools, and FUSTA with RAM caching works in a similar fashion to BioPython, which is reflected in their respective memory usage.

## 5 Results

FUSTA, by exposing an arbitrarily large multiFASTA file through a virtual arborescence, solves two of the main obstacles to their efficient handling.

First, by offering a read and write access to the content of any sequence in a multiFASTA file through standards UNIX file operations, it lets the user leverage any program or script of their choice while restricting memory usage to buffering only the needed sequences instead of the whole multiFASTA file.

Second, by automatically keeping track of the structure of the mounted multiFASTA file, it lets the user directly access or edit the sequences themselves without having to handle the parsing or buffering of multiFASTA files.

Thus, manual or automated operations on even large multiFASTA files are simplified.

## Supplementary Material

vbac091_Supplementary_Data

## References

[vbac091-B1] Cock P.J. et al (2009) Biopython: freely available python tools for computational molecular biology and bioinformatics. Bioinformatics, 25, 1422–1423. 19304878 10.1093/bioinformatics/btp163 PMC2682512

[vbac091-B2] Hunt M. (2012) Fastaq: Python3 scripts to manipulate fasta and fastq files*Github*. https://github.com/sanger-pathogens/Fastaq.

[vbac091-B3] Jackman S. (2012) fastascripts—manipulate fasta files*Github*. https://github.com/sjackman/fastascripts.

[vbac091-B4] Köster J. (2016) Rust-bio: a fast and safe bioinformatics library. Bioinformatics, 32, 444–446. 26446134 10.1093/bioinformatics/btv573

[vbac091-B5] Kryukov K. et al (2020) Sequence compression benchmark (SCB) database—a comprehensive evaluation of reference-free compressors for FASTA-formatted sequences. GigaScience, 9, giaa072. 32627830 10.1093/gigascience/giaa072 PMC7336184

[vbac091-B6] Li H. (2012) seqtk toolkit for processing sequences in fasta/q formats. *GitHub*. https://github.com/lh3/seqtk.

[vbac091-B7] Lipman D.J. , Pearson W.R. (1985) Rapid and sensitive protein similarity searches. Science, 227, 1435–1441. 2983426 10.1126/science.2983426

[vbac091-B8] Pearson W.R. , Lipman D.J. (1988) Improved tools for biological sequence comparison. Proc. Natl. Acad. Sci. USA, 85, 2444–2448. 3162770 10.1073/pnas.85.8.2444 PMC280013

[vbac091-B9] Shen W. et al (2016) SeqKit: a cross-platform and ultrafast toolkit for FASTA/Q file manipulation. PLoS One, 11, e0163962. 27706213 10.1371/journal.pone.0163962 PMC5051824

[vbac091-B10] Stajich J.E. et al (2002) The Bioperl toolkit: Perl modules for the life sciences. Genome Res., 12, 1611–1618. 12368254 10.1101/gr.361602 PMC187536

