# Supplementary material for: FUSTA: leveraging FUSE for manipulation of multiFASTA files at scale
Source: Bioinform Adv. 2022 Nov 29;2(1):vbac091. doi: 10.1093/bioadv/vbac091 (PMC9875552; doi:10.1093/bioadv/vbac091)
Supplement: vbac091_Supplementary_Data [file vbac091_supplementary_data.pdf]

FUSTA – Supplementary Material

Franklin Delehelle, Hugues Roest Crollius*

*Institut de Biologie de l'ENS (IBENS), Département de biologie, École normale supérieure, CNRS, INSERM, Université PSL; 46 rue d'Ulm, 75005 Paris, France

1 Use Case Examples

1.1 Introduction

Here, we present select examples of use cases covering the major features of FUSTA. All the following examples assume that the user mounted a multi-FASTA file of the GRCh38 human genome[1], for instance with:

```
fusta Homo_sapiens.GRCh38.dna.toplevel.fa
```

Once the file has been mounted (which might takes up to a few minutes depending on file size and disk access speed), the hierarchy of the created virtual files may be visualized with `tree -h fusta/`.

1.2 multiFASTA file content

Reading the `fusta/infos.txt` file, for instance with `cat fusta/infos.txt` will offer an overview of the content of the multiFASTA file, namely a list of sequences IDs (as defined in their respective FASTA headers) and sizes.

The `infos.csv` provide the same information, albeit in the CSV format, more suited to automated processing.

1.3 Extracting Subsequences

Extracting subsequences of various sizes from a large multiFASTA file is a very common operation, *e.g.*, for constituting variant databases (where a specific locus must be probed), to extract specific genes from full genome files, and so on.

FUSTA makes this manipulation straightforward by letting users directly extract any subsequence of the genome in a single operation through the `get/` folder. Although it appears empty, any access to a file following the `CHR:START-END` format would return the corresponding fragment.

For instance, reading the `get/chr17:18108706-18179802` file on the mounted human genome would return the sequence of the MYO15A gene, as if it were a free standing file containing only this sequence. With this mechanism, repeated access to random subsequences of a genome can be easily automatized, while remaining efficient thanks to the use of system-level file caches.

1.4 Accessing Sequences

The sequences contained in the mounted multiFASTA file can be accessed either in raw form (*i.e.*, only their representation in terms of nucleotides), or formatted as FASTA file (*i.e.*, including headers).

1.4.1 Raw Sequences

Raw sequences are available in the `seq/` folder, and can be accessed through the virtual file of the same name. For instance, `cat fusta/seq/chrY.fa` would output the sequence of the human Y chromosome.

[1] `http://ftp.ensembl.org/pub/release-106/fasta/homo_sapiens/dna/Homo_sapiens.GRCh38.dna.toplevel.fa.gz`

1.4.2 FASTA Sequences

FASTA sequences are available in the `fasta/` folder, and can be accessed through the virtual file of the same name. For instance, `cat fusta/fasta/chrMT.fa` would output the FASTA-formatted sequence of the mitochondria.

These virtual files can be used by other programs exactly as normal files; for instance, `blastn mydb.db -query fasta/chr2.fa` would use the human chromosome 2 sequence from the mounted GRCh38 as the query in a BLAST search.

1.4.3 Sequence Wrapping

Sequences are typically stored in two fashions: either as a single line, or as multiple lines wrapped to the same length. Although FUSTA transparently handles both cases, it may be used to convert from one to the other. For instance, a file containing wrapped sequences can be converted to single-line sequences with the following bash command:

```
# For all sequences
for i in $(find fusta-XYZ/seqs -type f); do
    echo "Processing $i"

    # Create a temporary file
    tmp_i=/tmp/$(basename $i)

    # Remove the newlines
    tr < $i -d '\n' > $tmp_i

    # Save the temporary file
    cat $tmp_i > $i
done
```

whereas the single-line to wrapped conversion can be done with:

```
# For all sequences
for i in $(find fusta-XYZ/seqs -type f); do
    # Create a temporary file
    tmp_i=/tmp/$(basename $i)

    # Remove newlines & split into 50 chars lines
    tr < $i -d '\n' | fold -w 50 > $tmp_i;

    # Save the temporary file
    cat $tmp_i > $i;
done
```

If the `sponge` utility is available, then explicit temporary files are not required and these scripts can be shortened respectively to:

```
for i in $(find fusta-XYZ/seqs -type f); do
    tr < $i -d '\n' | sponge $i;
done
```

and:

```
for i in $(find fusta-XYZ/seqs -type f); do
    tr < $i -d '\n' | fold -w 50 | sponge $i;
done
```

1.5 Adding, Removing & Renaming Sequences

1.5.1 Adding New Sequences

Adding new sequences to an existing multiFASTA files is straightforward through *e.g.*, the `cat` command; however, FUSTA offers this possibility for the sake of coherence: any FASTA file copied to the `append/` directory will be concatenated to the end of the mounted file.

For instance, `cp $HOME/more_sequences.fa append/` would append the sequences contained in the `more_sequences.fa` file to the mounted multiFASTA file.

1.5.2 Removing Sequences

Removing sequences and their headers from a multiFASTA file may prove useful in a few cases, *e.g.*, when extracting only the consensus sequences or the main chromosomes from a reference genome.

This can be done through the removal of virtual files in the `seqs/` folder. For instance, `rm seqs/chrMT.seq` would remove the mitochondrial sequence from the GRCh38 file; and `rm seqs/CHR_*` would purge the main file from alternate scaffolds.

1.5.3 Renaming Sequences

Although it is not necessarily a very common operation, renaming sequences may prove quite useful when changing nomenclatures. Like any other file, FUSTA virtual files can be renamed, *e.g.*, using the `mv` command, and this operation will rename the corresponding headers in the original multiFASTA file.

For instance, the infamous switch from the `ID.fasta` convention to the `chrID.fasta` one can be swiftly done through FUSTA with the following script: `cd fusta/seq; for i in *; do mv $i chr$i; done`

2 Ancillaries

2.1 Foreground/Background Modes

FUSTA can be run either in background mode, where it will detach from the shell and run in background until the corresponding mountpoint is unmounted, or in foreground mode (with `-D/--no-daemon`), where it will stay in the foreground until exit.

2.2 Caching Modes

FUSTA can leverage three methods of access to the underlying multiFASTA file, which can be chosen with the `--cache` parameter:

`mmap` (default, recommended) FUSTA leverages the memory mapping functionality of the OS to map the multiFASTA file in a memory region. This is the recommended mode for most uses as its leverage the system caches to their best;

`file` FUSTA will not explicitely use any caching mechanism, only reading the mounted file at the necessary positions whenever needed;

`memory` FUSTA will load the whole multiFASTA file in RAM. This provides the best performances, but requires as much free memory as the file size.

2.3 Operations Caching

To balance memory use and disk writes, FUSTA maintains a private memory cache for pending alterations to the mounted multiFASTA files. Those are (i) editions of sequences, (ii) appending of new sequences.

By default, the cache can grow up to 500MiB, at which point it is synchronized to the mounted file on disk. This value can be set through the `-C`/`--max-cache` parameter.
